# Genetic diversity and molecular evolution of human respiratory syncytial virus A and B

**DOI:** 10.1038/s41598-021-92435-1

**Published:** 2021-06-21

**Authors:** Jie-Mei Yu, Yuan-Hui Fu, Xiang-Lei Peng, Yan-Peng Zheng, Jin-Sheng He

**Affiliations:** College of Life Sciences and Bioengineering, Beijing Jiaotong University, Beijing, 100044 China

**Keywords:** Molecular evolution, Phylogenetics

## Abstract

Human respiratory syncytial viruses (RSVs) are classified into two major groups (A and B) based on antigenic differences in the G glycoprotein. To investigate circulating characteristics and phylodynamic history of RSV, we analyzed the genetic variability and evolutionary pattern of RSVs from 1977 to 2019 in this study. The results revealed that there was no recombination event of intergroup. Single nucleotide polymorphisms (SNPs) were observed through the genome with the highest occurrence rate in the G gene. Five and six sites in G protein of RSV-A and RSV-B, respectively, were further identified with a strong positive selection. The mean evolutionary rates for RSV-A and -B were estimated to be 1.48 × 10^–3^ and 1.92 × 10^–3^ nucleotide substitutions/site/year, respectively. The Bayesian skyline plot showed a constant population size of RSV-A and a sharp expansion of population size of RSV-B since 2005, and an obvious decrease 5 years later, then became stable again. The total population size of RSVs showed a similar tendency to that of RSV-B. Time-scaled phylogeny suggested a temporal specificity of the RSV-genotypes. Monitoring nucleotide changes and analyzing evolution pattern for RSVs could give valuable insights for vaccine and therapy strategies against RSV infection.

## Introduction

Human respiratory syncytial virus (RSV) is a major cause of serious lower respiratory tract illness in infants, young children and the elderly in both industrialized and developing countries^1^. It belongs to the genus *Orthopneumovirus*, family *Pneumoviridae*, with a nonsegmented, negative-sense RNA genome of approximately 15,200 nucleotides that contains 10 genes and encodes 11 proteins. Although RSV has a single serotype, it can be divided into two antigenic groups: A and B, according to the epitope differences mainly in the attachment glycoprotein (G)^2,3^. G protein is the most variable protein^4^, it has two hypervariable regions flanking the highly conserved central region, and the hypervariable regions contain most antigenic differences both in the inter- and intra-group of the virus, with the C-terminal or the second hypervariable region encompasses the strain-specific epitopes^5^, commonly sequenced to determine the genotype (strain or clade) or investigate genetic diversity of RSV strains^6–8^.

RSV-A group can be further classified into 9 genotypes (GA1-GA7, SAA1 and NA1)^9^, while RSV-B group is subdivided into at least 32 genotypes: BA1-14^10–12^, GB1-GB5^13,14^, SAB1-4^15^, URU1-2^16^, NZB1-2^17^, BA-CCA, BA-CCB, BA-C^18^, CBB^19^ and CB1^20^. It was observed that RSV from both groups and distinct clades can co-circulate locally in successive years, but alternates in the predominance of group A and Bas well as the corresponding clades in 1- or 2-year cycles^8,21^. Of note, although the pattern of alternative prevalence between RSV-A and RSV-B remains, a single RSV-A NA1 genotype and a single RSV-BBA9 genotype were predominant in the population since 2011 and 2014, respectively^9^. Both the emergence of new genotypes and the disappearance of the earlier genotypes highlight the ongoing evolution of the RSV.

Some unique genetic modifications in RSV G gene have been identified, including a 72 nucleotide duplication in A group and a 60 nucleotide duplication in B group, designated as the ON1 (proposed as one of the lineages of NA1 genotype) and BA genotype, respectively^9,22,23^. The novel RSV strains with G duplications spread rapidly worldwide and exhibit a fitness advantage^24,25^. In contrast, the fusion glycoprotein (F), mediating both virus-cell membrane fusion and bindings to Toll-like receptor 4 (TLR-4)^26^, is highly conserved antigenically and genetically and it is the main target of many anti-RSV monoclonal antibodies (mAbs) and vaccines in clinical development^27^. However, the amino acid changes in the neutralizing antigenic sites in RSV F, especially from RSV-B isolates were reported^25^; also, amino acid changes were found in viral polymerase and M2-1^28^. Generally, it is presumed that G protein is more diversified than all the other proteins, but the high degrees of RSV G diversity are not entirely driven by the evasion of adaptive host immunity^29^.

Mutation and recombination are the major factors affecting the molecular evolution of RNA viruses^30^. It was strongly suggested that selective pressures are continuing to drive the evolution of RSV, genetically and phenotypically^23,31^. In this study, we performed a systemic evolutionary analysis upon globally updated RSV complete sequences released in public website that spanning more than 40 years to determine the genetic diversity, molecular evolution and phylogeny of the RSVs.

## Results

### Recombination analysis on representative RSV sequences

**Figure 1:**
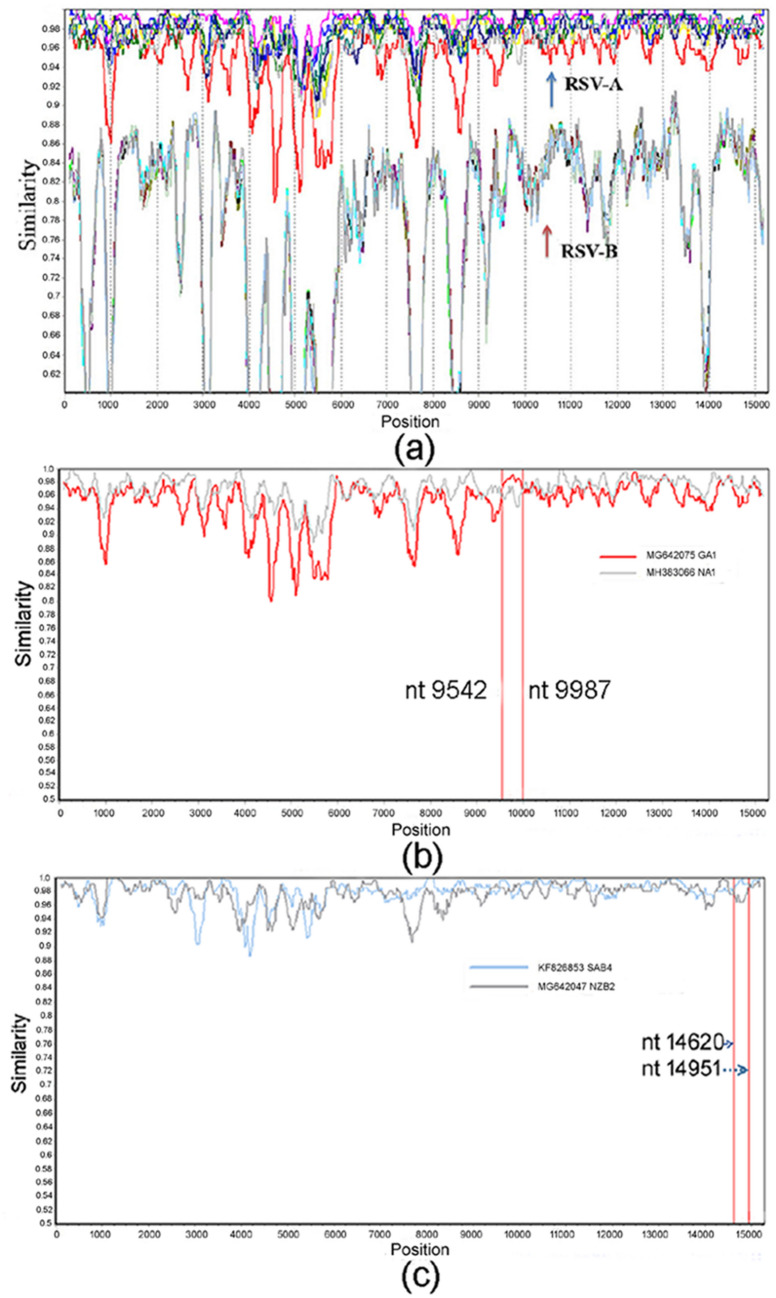
Recombination analysis in RSVs by using representative subtypes/genotypes. (**a**) RSV-A and RSV-B: no recombination event was observed; (**b**) RSV-A and (**c**) RSV-B: potential recombination breakpoints in the intragroups were marked in red lines and the positions were annotated.

Nineteen complete genome sequences that representing different genotypes of RSV A and B were aligned together, nine for RSV-A and ten for RSV-B. RSV-A (KJ723483, GA6 genotype) was used as query sequence to analyze the potential recombination events of RSVs. Results showed that no recombination occurred between the group of RSV-A and RSV-B (Fig. 1a); however, in the intragroups, some sequences showed an evidence of mosaicism (Fig. 1b,c), indicating involvement in homologous recombination with identifiable parental.

### Genetic diversities of RSVs

Following the performance of single nucleotide polymorphism (SNP) calling in the genes of both RSV-A and -B, a large number of SNPs were found in their genomes, indicating a high level of genetic diversity of RSVs. Among the ten genes of the virus, G gene has the highest SNP occurrence rate, with over 28 SNPs per 100 nt on average, while for the other genes, the SNP occurrence rates were much lower, ranged from 5 to 11%. Overall, it was shown that the number of SNPs in the RSV-A was larger than that in RSV-B, with G gene as an exception, its SNP occurrence rate of RSV-B was higher than that of RSV-A (Table 1). The dN to dS ratio for each gene was also determined in the study. It was shown that the ratios of the G genes for RSV-A and -B were both > 1, indicating a strong positive selection in this gene. The ratios for all the other genes were < 1, indicating a negative selection pressure in these genes (Table 1).

**Table 1:** Number of SNPs and ratios of dN/dS for each genes of theRSV-A and -Bgenomes. Numbers in bold are positive selection sites by both the MEME and FUBAR methods.

Gene	Group	Length (bp)	No. of SNP	No. of SNP per 100 nt	dN/dS	Selection type
NS1	A	420	29	6.9	0.088	Negative
	B	420	27	6.4	0.373	Negative
NS2	A	375	41	10.9	0.152	Negative
	B	375	32	8.5	0.312	Negative
N	A	1176	100	8.5	0.049	Negative
	B	1176	59	5.0	0.087	Negative
P	A	726	60	8.3	0.101	Negative
	B	726	55	7.6	0.100	Negative
M	A	771	73	9.5	0.032	Negative
	B	771	57	7.4	0.063	Negative
SH	A	195	19	9.7	0.274	Negative
	B	198	15	7.6	0.632	Negative
G	A	969	272	**29.6**	**1.663**	**Positive**
	B	984	282	**33.8**	**1.695**	**Positive**
F	A	1725	169	9.8	0.148	Negative
	B	1725	124	7.2	0.162	Negative
M2	A	826	84	10.2	0.603	Negative
	B	826	71	8.6	0.562	Negative
L	A	6498	519	8.0	0.174	Negative
	B	6510	405	6.2	0.170	Negative

### Site-specific selection analysis of the RSV G gene

As were observed above, the average ratios of nonsynonymous to synonymous nt substitutions in the G genes of RSV A and B were indicative for positive selection, we further examined the coding sequences of different isolates to detect the codons that provide stronger evidence of purifying or diversifying selection in the gene. It was shown that both negative and positive selection happened in the G gene, with 63 and 65 sites were respectively defined as negative selection in RSV-A and RSV-B by FUBAR method, while 11 sites in RSV-A and 9 sites in RSV-B were defined as positive selection (by at least one of the two methods), among which five sites in RSV-A and six sites in RSV-B had the strongest support, by both method MEME and FUBAR methods (Table 2). Two and three positively selected sites respectively in RSV-A and RSV-B were located in the first hyper-variable region (amino acid positions 94, 115 in RSV-A and 77, 115, 133 in RSV-B), while seven and six positively selected sites were located in the carboxy-terminal third of the G ectodomain.

**Table 2:** Positive selection sites in G gene of RSV-A and -B.

RSV group	Methods	Positive sites
A (63 negative sites)	MEME*	15, **94**, **115**, **196**, 206, 221, **286**, 298, **314**
	FUBAR^#^	**94**, **115**, **196**, 237, 262, **286**, **314**
B (65 negative sites)	MEME*	77, 115, **133**, **219**, **238**, **278**, **289**, **295**, 311
	FUBAR^#^	**133**, **219**, **238**, **278**, **289**, **295**

Numbers in bold are positive selection sites by both the MEME and FUBAR methods.

“*”: p value ≤ 0.05; “^#^”: poster probability of 0.9.

### Evolutionary rate estimation

**Figure 2:**
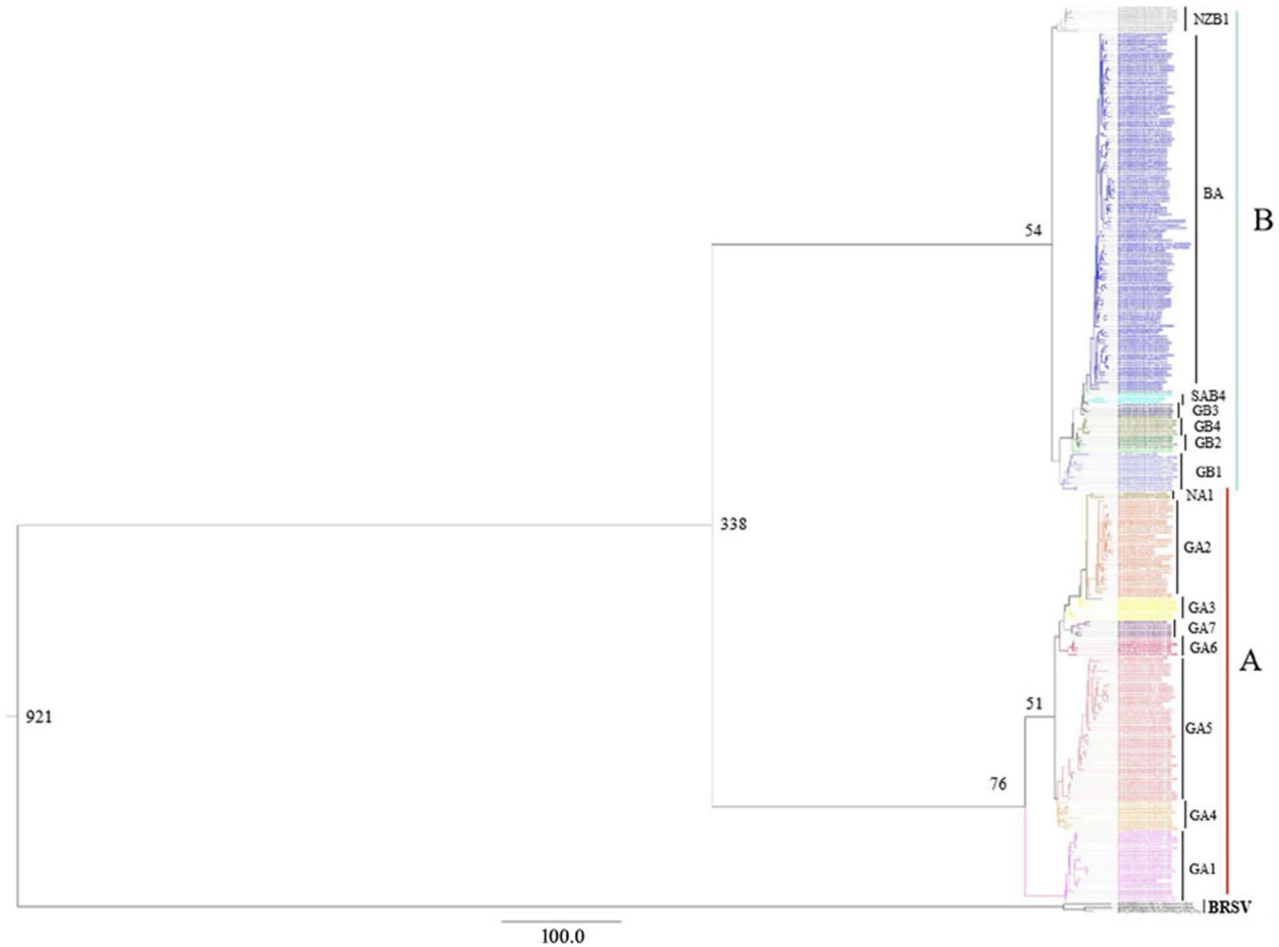
Bayesian Markov Chain Monte Carlo tree of the complete G genes of RSV-A, RSV-B and BRSV. The estimated time of the MRCA of the major nodes and the names of different viral clades were marked in the graph. The results showed that RSV-A and RSV-B diverged from their common ancestor 338 years ago.

To determine the evolutionary relationship between the different RSV strains, a Bayesian MCMC estimation of the time of the most recent common ancestor (MRCA) was performed. Results suggested that the uncorrelated lognormal relaxed molecular clock fit the data best. Under the best-fit model, the totally evolutionary rate for RSVs was calculated as 2.03 × 10^–3^ nucleotide substitutions/site/year, with 95% HPD interval as 1.46 × 10^–3^ to 3.06 × 10^–3^. The evolutionary rate for RSV-A was calculated as 1.48 × 10^–3^ nucleotide substitutions/site/year (95% HPD interval: 1.27 × 10^–3^ to 1.71 × 10^–3^), while the evolutionary rate for RSV-B was 1.92 × 10^–3^ (95% HPD interval: 1.69 × 10^–3^ to 2.16 × 10^–3^). The MRCA of RSV and BRSV was estimated to be around 921 years ago, while the MRCA of RSV-A and RSV-B dates back to 338 years ago. The earliest genotypic differentiation for RSV-A and RSV-B was 76 and 54 years ago, i.e. in the year of 1943 and 1965, respectively (Fig. 2).

### Population dynamic analyses

**Figure 3:**
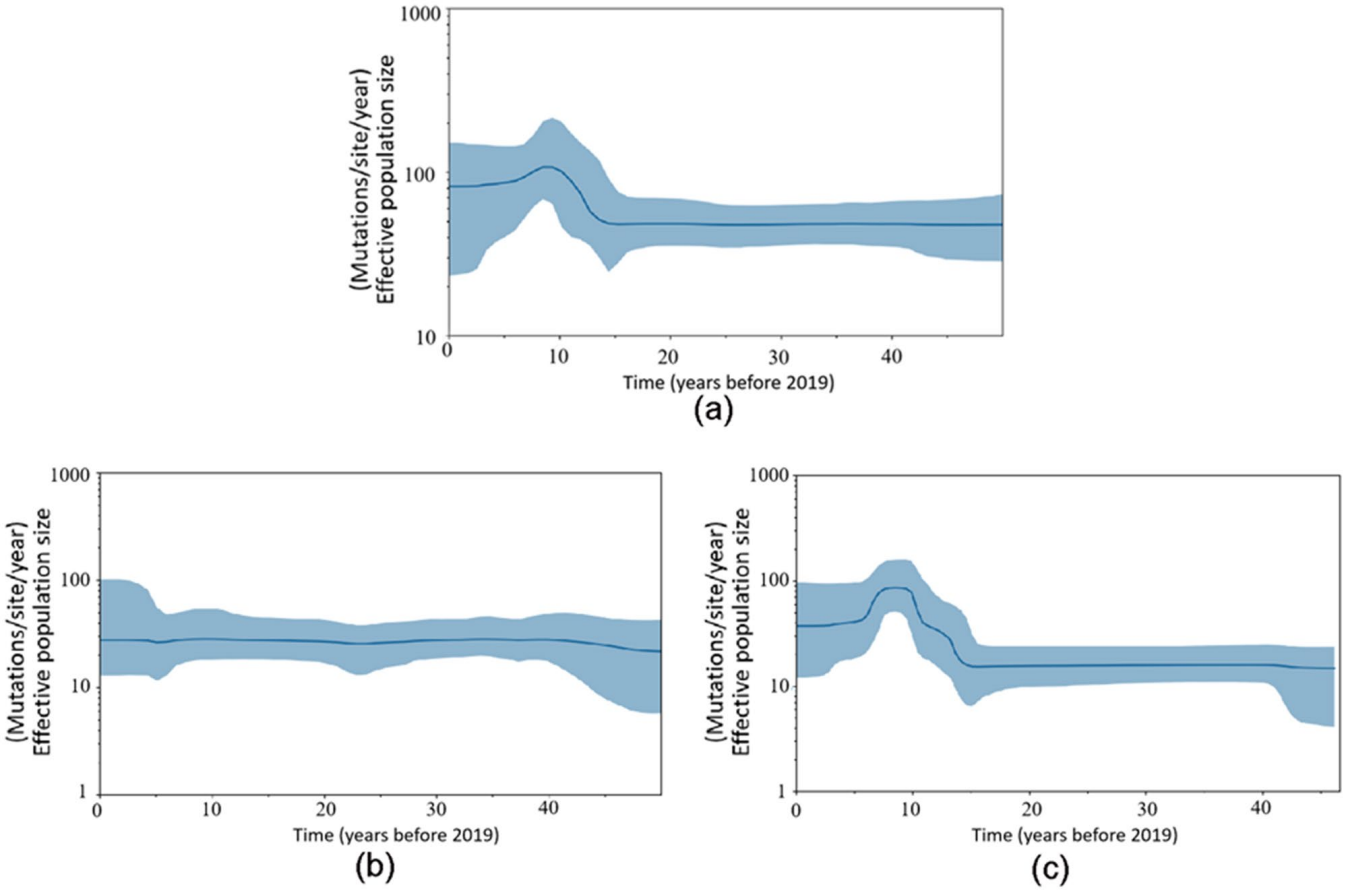
Population dynamics of the G gene for the RSV population using Bayesian skyline plot analyses. (**a**) both the RSV-A and RSV-B strains; (**b**) RSV-A; (**c**) RSV-B. The thick solid lines indicate mean effective population sizes; blue shadings stand for 95% HPD.

We estimated the effective population sizes of the prevalent strains of RSV using Bayesian skyline plot analyses. It was shown that the total RSVs witnessed a constant population size until around 15 years ago, when a sharp increase in population size lasted for about 5 years, followed by a short plateau period of 1–2 years before going down and became stable again (Fig. 3a). The fluctuation of the effective population size of RSV-B witnessed the same pattern with the total RSV population (Fig. 3c). The effective population size of RSV-A witnessed a persistent population size through the time axis (Fig. 3b).

### Time-scaled phylogeny reconstruction

**Figure 4:**
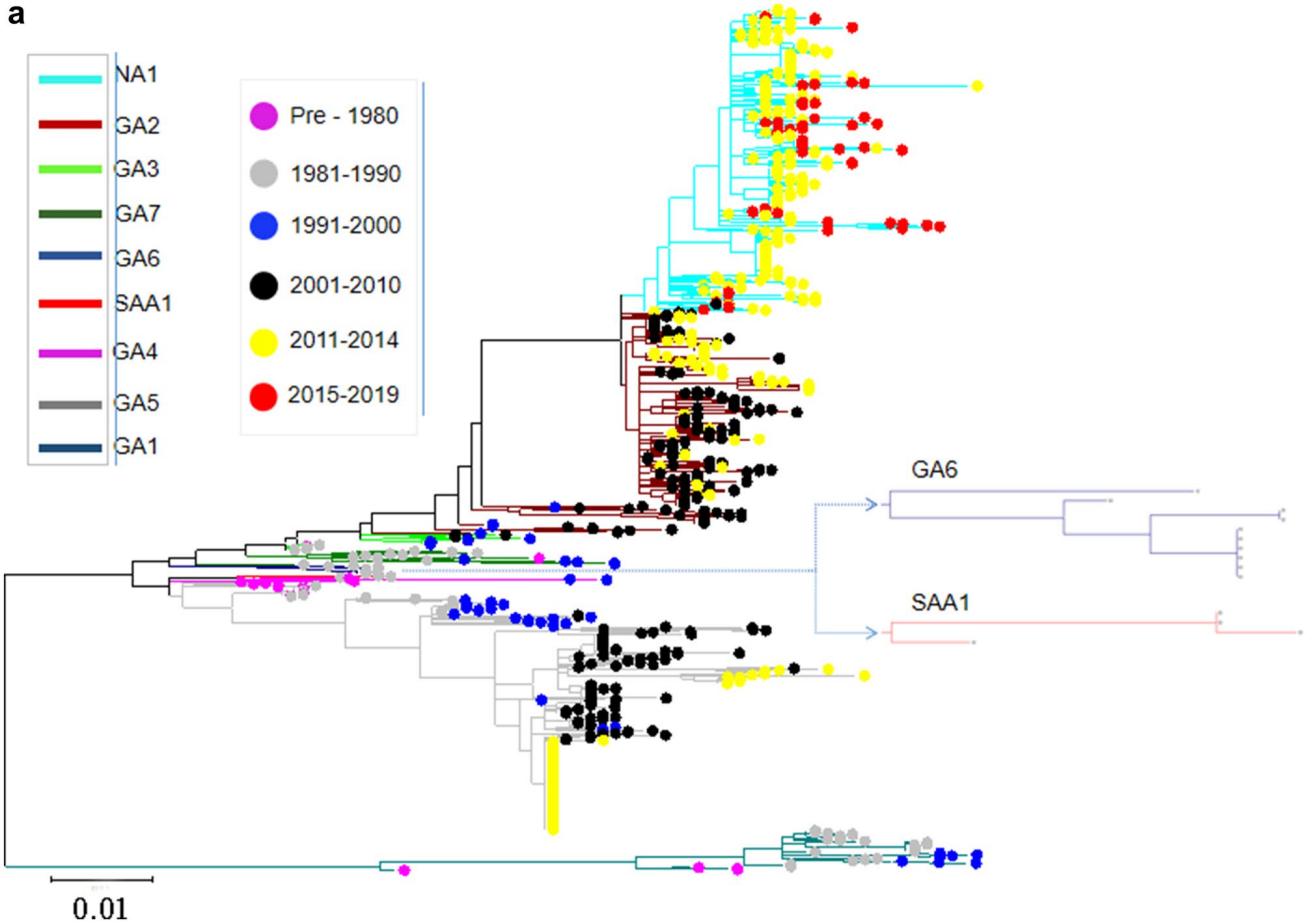

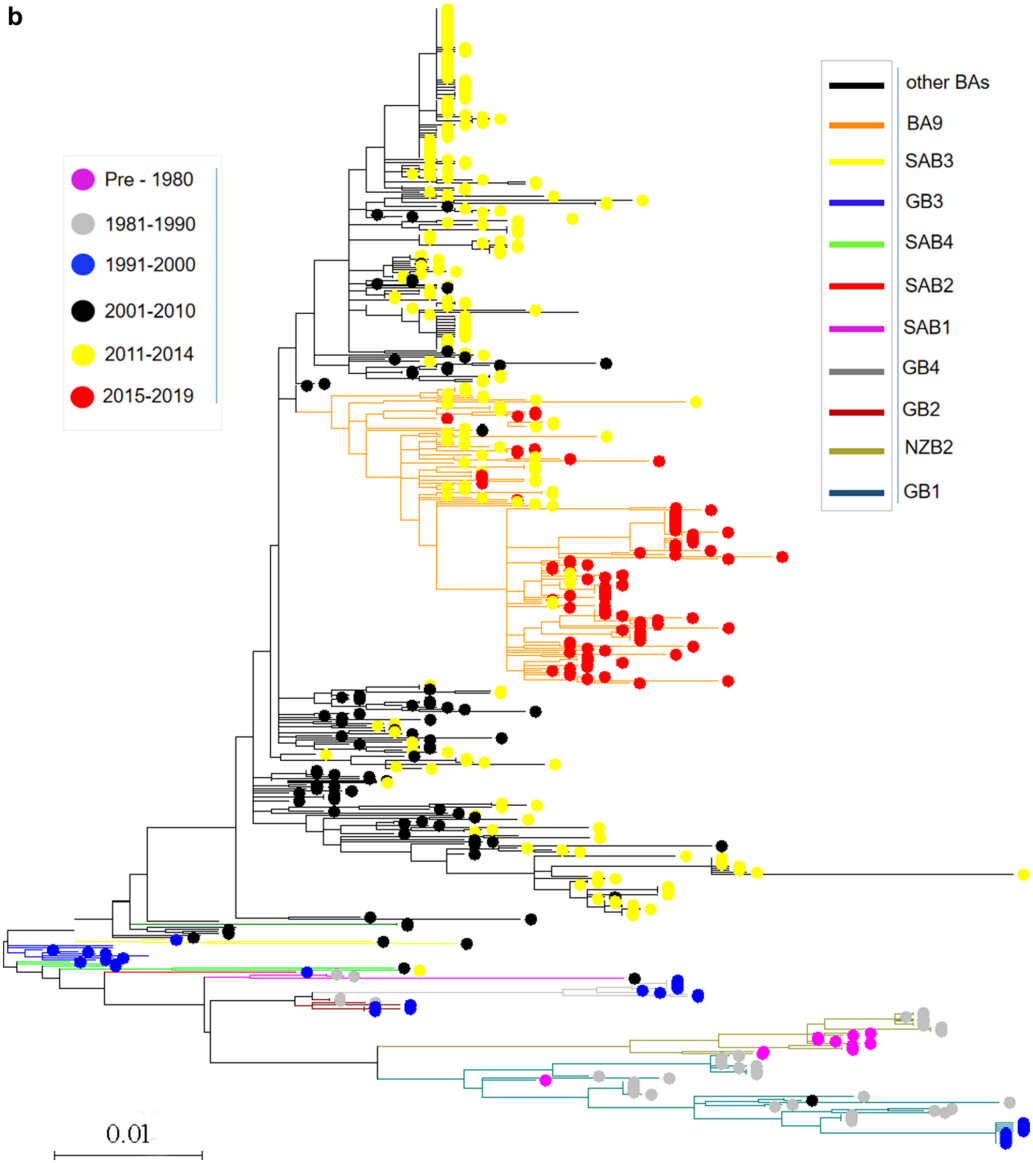
Temporal distribution of evolutionary clusters in the unrooted phylogenetic tree based on the RSV G gene sequence alignments using maximum likelihood method. (**a**) RSV-A: the invisible GA6 and SAA1 were further enlarged on the right side of the figure; (**b**) RSV-B. Dots with different color indicate sequences from different time, and lines with different color indicate different subtypes.

To trace the spreading history of RSV, an unrooted maximum likelihood tree was built using the aligned complete G gene. The results suggested that the prevalence of the genotypes for both RSV-A and RSV-B was temporal specific. For RSV-A, the genotype with the longest epidemic duration was GA5, but it disappeared since 2015, SAA1, GA7 and GA6 genotypes were only prevalent in the 1980s and before, GA1 and GA4 genotypes were prevalent before 2000, GA3 prevalent before 2010, while GA2 was prevalent between the year of 2001 and 2014, since 2015, the only prevalent genotype was NA1 (Fig. 4a). For RSV-B, the mainly prevalent genotypes in the early stage (1970s and 1980s) were GB1, GB2, GB4 and NZB2, SAB1-4 genotypes were prevalent in 1990s to 2000s, BA genotypes (BA1-BA14) was most predominant in the last 20 years, and since 2015, the only prevalent genotype was BA9 (Fig. 4b).

## Discussion

RSV is a large family containing two different antigenic groups, further clustered into distinct genotypes. The variability and evolutionary dynamics of the circulating strains of RSV-A and -B in the past 50 years worldwide has been clarified in this study. Generally, RNA viruses maintain extensive genetic variability through which rapid evolution can occur. Recombination is an important way for viral evolution both in positive- and negative-sense RNA viruses such as coronaviruses, astroviruses and influenza A virus^32–34^, it involves the generation of chimeric nucleic acid molecules consisting of components from different parental viruses. However, recombination events yielding different progeny have not been reported in RSV except one study involving the potential recombination during mixed infection in vitro^35^. Our recombination analysis on representatives of RSV-A and -B genotypes in this study also showed there was no recombination event occurred between the groups, but potential small fragment recombination events were observed in the intra-groups of both RSV-A and RSV-B. However, since continuous changes in temporal distribution of RSV subtypes/genotypes have been recorded^9^, co-infection with different subtypes/genotypes in the same host was rarely reported^20,36,37^. Therefore, we are tend to believe that the potential intragenic recombination events in this study were not true, as current recombination detection programs have not enabled a clear distinction between recombination or genetic drift in the reported breakpoints of closely related strains.

Mutation is another important factor in the evolution of RNA virus. The mutations occurred by chance for lacking of proof-reading capabilities of RNA-dependent RNA polymerase of the virus, which shape quasispecies to allow rapid adaptation of the virus to the changed environment such as host’s immune pressure. In this study, comparison of mutation rates for different genes of RSV showed that SNP occurrence rate of the G gene in both RSV-A and -B was much higher than that of other genes, and positive selection was only found in the G gene compared with negative selections in all the other genes. This phenomenon suggested that the pretty high genetic diversity G gene reflects the impact of host immune responses in the epidemic cycle, whereas purifying selection in the remaining genes reflects avoiding deleteriously mutations and favoring the optimization of viral replication and transmission. Substitutions, insertions, deletions, duplications, stop codon usage change and frame shift mutation all involved in the variability of G gene and may be related to its escape from the protection of the host neutralizing antibodies. Our study showed that except for G gene, the mutation frequencies of other genes were higher in RSV-A than in RSV-B, which may be attributed by a wider prevalence of RSV-A^38,39^. The mutation frequency of the G gene of RSV-B was higher than that of RSV-A, which may suggested a broad antigenic diversity in RSV-B, and this result was consistent with the phenomenon that RSV-A reacted with all the antibodies, whereas RSV-B showed different epitope characteristics in G gene in previous study in the very early time^3^.

Although G gene exclusively harbors the most diversified mutations selected partially as response to host immunological pressures^29^, adaptive evolution is able to affect only certain sites of the gene because of strong functional constraints. In this study, the number of positive selection sites in G gene was less than those in the previous studies^40,41^, which may be caused by strict standards of P value (0.05 versus 0.1) and poster probability (0.9 versus 1.0). However, a similar distribution of the positive sites was observed: a small part of sites located in the first hypervariable region and a large part located in the C-terminal third of the ectodomain of the G, an important determinant of RSV evolution, but whether the immune pressure on G protein drives the selection completely needs to be further explored, since the random genetic drift following potential bottleneck effect rather than immune selection is still a challenge confronted or a point to be excluded: on one hand, the C-terminal strain specific epitopes are poor or secondary as neutralizing antigens and contribute much less in inducing the potent neutralizing antibody compared with those highly conserved neutralizing epitopes in F and G proteins; on the other hand, a recent advances in the vaccine development showed a robust and durable neutralizing antibody responses, induced by recombinant adenovirus encoding prefusion F (pre-F), can confer cross-protection against RSV-A and -B infection in old healthy people^42^, suggesting the short-lived and low level of neutralizing antibody response against F protein following RSV natural infection is responsible for the transmission and evolution of diversified RSV. In other words, the highly diverse and positively selected domains in G protein are not the main contributor to the evolution of RSV. It may be more easily accepted to notice the fact that different from the positively selected and constantly changed neutralizing epitopes in hemagglutinin (HA) of influenza virus^43^, the strong neutralizing epitopes of RSV exist either in the conserved F protein or in the highly conserved central domain of G protein. In sharp contrast, the positively selected antigens in G protein is much poor in inducing neutralizing antibody. Therefore, it was speculated that if the mutations from the positive selection sites on the G gene do have some contributions biologically and immunologically to the escape and evolution, it should be the secondary to be taken into account.

The evolutionary rates estimated for the genes of RSV-A and -B in the present study were 1.48 × 10^–3^ and 1.92 × 10^–3^ nucleotide substitutions/site/year, respectively, which were close to the previously estimated rates that approximately ranged from 1.83 × 10^–3^ to 4.68 × 10^–3^ substitutions/site/year in RSV-A and 1.95 × 10^–3^ to 5.89 × 10^–3^ substitutions/site/year in RSV B^44,45^. Notably, it has been suggested that the evolutionary rate of G genes were about 10 times faster than that of the complete genomes and F genes of RSV^40,46,47^. The possible explanation for the faster mutation rate of G genes was that this gene may be under a strong selective pressure from host immune defense which was stronger than the F and other genes of the RSVs, and can lead to variants that may alter the pathogenicity and fitness^48^. Based on the evolutionary rates, it was estimated that the MRCA for the tree root can date back to the year of 1943 for RSV-A and 1965 for RSV-B, which was comparable to the results in the previous studies^41,44,45^. The calculations in our study suggested that the divergence of RSV-A and -B viruses occurred approximately 338 years ago, indicating the MRCA of the two groups can date back to the year of 1681.

The population dynamics showed that RSV-A witnessed a constant population size through the time axis, while an obvious increase in population size about 15 years ago (in 2004) was observed in RSV-B, which may be caused by the application of the next generation sequencing technology at the time that had detected a large number of RSV sequences. However, the obvious decrease in RSV-B detected about 6–7 years ago (in 2012–2013) might result from dying out of some genotypic lineages, as the temporal dynamic analysis in this study also showed that different BA types were detected in 2011–2014, while since 2015, only BA9 was observed. Because of RSV-A being more prevalent than RSV-B^17,38,39,49^, the factors affecting RSV-B population size have little influence on RSV-A. The change trend of the population size of total RSV was consistent with that of RSV-B, which suggested that changes in effective population size were group specific and the recent change in RSV-B may be the source of the observed change of the whole RSV family.

Our estimation of the temporal dynamics suggested that different new genotypes of both RSV-A and -B appear periodically and tend to be the predominated circulating strains. For RSV-B, GB1 was the earliest prevalent genotype, while the BA was the only prevalent genotype in the last 20 years, and since 2015, the only prevalent genotype was BA9; while for RSV-A, the longest duration of the epidemic was GA5 genotype, and since 2011, the only prevalent genotype was NA1. It is worth noting that the most prevalent genotypes for RSV-A and-B in the last 10 years was NA1 (especially ON1 lineage) and BA (BA1-14), respectively. A 72-nt and a 60-nt nucleotide duplication was respectively found in the C-terminal region of the G protein of ON1 and BA genotypes, which made the virus had a fitness advantage to the host, and the corresponding genotypes soon became the predominant strains in the world^15,22,50^.

In summary, this study provides data for genetic diversity and molecular evolution with RSVs circulated in the world. Since the genetic diversity of viral genome and the variability of G glycoprotein play a significant role in RSV pathogenicity by allowing immune evasion, monitoring changes and analyzing evolution pattern in the sequences of both groups could give useful insights for future vaccine and therapy strategies of RSV.

## Methods

### Genome sequence dataset retrieval

Complete genome sequences of the RSVs with clear isolation time and local were downloaded from the Virus Pathogen Database and Analysis Resource (ViPR, https://www.viprbrc.org/), among which 558 sequences were RSV-B, while 956 were RSV-A. Full-length genome sequences of the bovine RSV (BRSV) were downloaded from the NCBI GenBank Database (https://www.ncbi.nlm.nih.gov/genbank). All the genomic sequences were aligned by MAFFT 7 (online version: https://mafft.cbrc.jp/alignment/software/).

### Recombination analysis

To detect whether any potential recombination occurred in the inter- or intra-groups of RSVs, the whole-genome sequences from their representatives of different genotypes of RSV A and B were aligned and analyzed by Simplot software (version 3.5.1) using the boot scanning method, with neighbor joining algorithm as 100 pseudoreplicates.

### Calculation of genetic diversity

SNP calling of the genes of RSVs was done on the ViPR (https://www.viprbrc.org/). Diversities of the different genes that encode the RSV proteins were quantified as the mean genetic distance calculated for the pairs of nucleotide sequences using MEGA software (version 7). The ratio of nonsynonymous substitutions per nonsynonymous site (dN) and synonymous substitutions per synonymous site (dS) were calculated using the method of Nei and Gojobori with the Jukes-Cantor correction for multiple substitutions, conducted using MEGA software (version 7). The dN/dS ratio is an indicator of the strength of positive (> 1) or negative (< 1) or neutral (= 1) selection pressure on a viral variant.

### Site selection pressure on the RSV G gene

Estimation of diversifying and purifying selection sites for the aligned G gene sequences was developed by Datamonkey (online version: http://www.datamonkey.org/). The non-synonymous and synonymous substitution rates were calculated for each codon of G using the mixed-effects model of evolution (MEME) and fast unbiased Bayesian approximation (FUBAR). The significance level was set at 0.05.

### Evolutionary rate estimates

To precisely estimate the substitution rate of G gene in populations, a Bayesian Markov Chain Monte Carlo (MCMC) approach was implemented using BEAST software (version 2.6.3). Iqtree package (version 1.6.12) was used to identify the optimal evolutionary model. After computation, the results were analyzed and presented by Tracer v.1.7.1. The effective sample size (ESS) values for all the estimated parameters in the MCMC analyses in the study were > 200. Statistical uncertainty in parameter values of the data was reflected by the 95% highest probability density (HPD) values. Tree annotator software was used to run the qualified file, and then FigTree (version 1.4.2) was used to further show the tree structure.

### Bayesian skyline plot

To clarify the effective population size of the RSV prevalent variants, Bayesian skyline plot analyses (BSP) were characterized using BEAST software (version 2.6.3). GTR+ Γ with Relaxed Clock Log Normal and constant growth demographic population dynamic models were used as the fittest model to the dataset. The MCMC analyses were performed 50 million generations and sampling every 2000 generations with 20% burn-in. The BSP was analyzed using Tracer software.

### Time-scaled phylogeny reconstruction

The aligned sequences for RSV-A and -B were used for phylogeny analysis. The phylogenetic tree was performed in MEGA 7.0.26 software using Maximum likelihood method with Kimura 2-parameter substitution model. Different genotypes and circulating time were marked.

## Supplementary Information

Supplementary Information.

## Data Availability

The datasets analyzed during this study are included in this published article in Supplementary Information files.

## References

[1] Shi T (2017). Global, regional, and national disease burden estimates of acute lower respiratory infections due to respiratory syncytial virus in young children in 2015: A systematic review and modelling study. Lancet.

[2] Anderson LJ (1985). Antigenic characterization of respiratory syncytial virus strains with monoclonal antibodies. J. Infect. Dis..

[3] Mufson MA, Orvell C, Rafnar B, Norrby E (1985). Two distinct subtypes of human respiratory syncytial virus. J. Gen. Virol..

[4] Johnson PR, Spriggs MK, Olmsted RA, Collins PL (1987). The G glycoprotein of human respiratory syncytial viruses of subgroups A and B: Extensive sequence divergence between antigenically related proteins. Proc. Natl. Acad. Sci. USA.

[5] Martinez I, Dopazo J, Melero JA (1997). Antigenic structure of the human respiratory syncytial virus G glycoprotein and relevance of hypermutation events for the generation of antigenic variants. J. Gen. Virol..

[6] Galiano MC (2005). Intragroup antigenic diversity of human respiratory syncytial virus (group A) isolated in Argentina and Chile. J. Med. Virol..

[7] Reiche J, Schweiger B (2009). Genetic variability of group A human respiratory syncytial virus strains circulating in Germany from 1998 to 2007. J. Clin. Microbiol..

[8] Peret TC, Hall CB, Schnabel KC, Golub JA, Anderson LJ (1998). Circulation patterns of genetically distinct group A and B strains of human respiratory syncytial virus in a community. J. Gen. Virol..

[9] Munoz-Escalante JC (2019). Respiratory syncytial virus A genotype classification based on systematic intergenotypic and intragenotypic sequence analysis. Sci. Rep..

[10] Dapat IC (2010). New genotypes within respiratory syncytial virus group B genotype BA in Niigata, Japan. J. Clin. Microbiol..

[11] Bashir U (2017). Molecular detection and characterization of respiratory syncytial virus B genotypes circulating in Pakistani children. Infect. Genet. Evol..

[12] Gimferrer L (2019). Virological surveillance of human respiratory syncytial virus A and B at a tertiary hospital in Catalonia (Spain) during five consecutive seasons (2013–2018). Future Microbiol..

[13] Peret TC (2000). Circulation patterns of group A and B human respiratory syncytial virus genotypes in 5 communities in North America. J. Infect. Dis..

[14] Ren L, Xiao Q, Zhou L, Xia Q, Liu E (2015). Molecular characterization of human respiratory syncytial virus subtype B: A novel genotype of subtype B circulating in China. J. Med. Virol..

[15] Arnott A (2011). A study of the genetic variability of human respiratory syncytial virus (HRSV) in Cambodia reveals the existence of a new HRSV group B genotype. J. Clin. Microbiol..

[16] Blanc A, Delfraro A, Frabasile S, Arbiza J (2005). Genotypes of respiratory syncytial virus group B identified in Uruguay. Arch. Virol..

[17] Matheson JW (2006). Distinct patterns of evolution between respiratory syncytial virus subgroups A and B from New Zealand isolates collected over thirty-seven years. J. Med. Virol..

[18] Zheng Y (2017). Prevailing genotype distribution and characteristics of human respiratory syncytial virus in northeastern China. J. Med. Virol..

[19] Baek YH (2012). Prevalence and genetic characterization of respiratory syncytial virus (RSV) in hospitalized children in Korea. Arch. Virol..

[20] Cui G (2013). Genetic variation in attachment glycoprotein genes of human respiratory syncytial virus subgroups a and B in children in recent five consecutive years. PLoS ONE.

[21] Waris M (1991). Pattern of respiratory syncytial virus epidemics in Finland: Two-year cycles with alternating prevalence of groups A and B. J. Infect. Dis..

[22] Eshaghi A (2012). Genetic variability of human respiratory syncytial virus A strains circulating in Ontario: A novel genotype with a 72 nucleotide G gene duplication. PLoS ONE.

[23] Trento A (2015). Conservation of G-protein epitopes in respiratory syncytial virus (Group A) despite broad genetic diversity: Is antibody selection involved in virus evolution?. J. Virol..

[24] Agoti CN, Otieno JR, Gitahi CW, Cane PA, Nokes DJ (2014). Rapid spread and diversification of respiratory syncytial virus genotype ON1, Kenya. Emerg. Infect. Dis..

[25] Bin L (2019). Emergence of new antigenic epitopes in the glycoproteins of human respiratory syncytial virus collected from a US surveillance study, 2015–17. Sci. Rep..

[26] Lamb RA, Parks GD, Knipe DM, Howley PM, Cohen JI, Griffin DE, Lamb RA, Martin MA, Racaniello VD, Roizman B (2013). Respiratory syncytial virus and metapneumovirus. Fields Virology.

[27] Bates JT (2016). Immunogenicity and efficacy of alphavirus-derived replicon vaccines for respiratory syncytial virus and human metapneumovirus in nonhuman primates. Vaccine.

[28] Otieno JR (2018). Whole genome analysis of local Kenyan and global sequences unravels the epidemiological and molecular evolutionary dynamics of RSV genotype ON1 strains. Virus Evol..

[29] Tan L (2013). The comparative genomics of human respiratory syncytial virus subgroups A and B: Genetic variability and molecular evolutionary dynamics. J. Virol..

[30] Sanjuan R, Nebot MR, Chirico N, Mansky LM, Belshaw R (2010). Viral mutation rates. J. Virol..

[31] Zlateva KT, Lemey P, Vandamme AM, Van Ranst M (2004). Molecular evolution and circulation patterns of human respiratory syncytial virus subgroup a: Positively selected sites in the attachment g glycoprotein. J. Virol..

[32] Bean WJ, Cox NJ, Kendal AP (1980). Recombination of human influenza A viruses in nature. Nature.

[33] Rehman SU, Shafique L, Ihsan A, Liu Q (2020). Evolutionary trajectory for the emergence of novel coronavirus SARS-CoV-2. Pathogens.

[34] Yu JM (2020). Complete genome of a novel recombinant human astrovirus and its quasispecies in patients following hematopoietic stem cell transplantation. Virus Res..

[35] Spann KM, Collins PL, Teng MN (2003). Genetic recombination during coinfection of two mutants of human respiratory syncytial virus. J. Virol..

[36] Moreira FB (2017). Molecular characterization and clinical epidemiology of human respiratory syncytial virus (HRSV) A and B in hospitalized children, Southern Brazil. J. Med. Virol..

[37] Yu X (2015). Human respiratory syncytial virus in children with lower respiratory tract infections or influenza-like illness and its co-infection characteristics with viruses and atypical bacteria in Hangzhou, China. J. Clin. Virol..

[38] Park E (2017). Molecular and clinical characterization of human respiratory syncytial virus in South Korea between 2009 and 2014. Epidemiol. Infect.

[39] Madi N, Chehadeh W, Asadzadeh M, Al-Turab M, Al-Adwani A (2018). Analysis of genetic variability of respiratory syncytial virus groups A and B in Kuwait. Arch. Virol..

[40] Tan L (2012). Genetic variability among complete human respiratory syncytial virus subgroup A genomes: Bridging molecular evolutionary dynamics and epidemiology. PLoS ONE.

[41] Martinelli M (2014). Phylogeny and population dynamics of respiratory syncytial virus (Rsv) A and B. Virus Res..

[42] Williams K (2020). Phase 1 safety and immunogenicity study of a respiratory syncytial virus vaccine with an adenovirus 26 vector encoding prefusion F (Ad26.RSV.preF) in adults aged ≥60 years. J. Infect. Dis..

[43] Fitch WM, Leiter JM, Li XQ, Palese P (1991). Positive Darwinian evolution in human influenza A viruses. Proc. Natl. Acad. Sci. USA.

[44] Zlateva KT, Lemey P, Moes E, Vandamme AM, Van Ranst M (2005). Genetic variability and molecular evolution of the human respiratory syncytial virus subgroup B attachment G protein. J. Virol..

[45] Pretorius MA (2013). Replacement and positive evolution of subtype A and B respiratory syncytial virus G-protein genotypes from 1997–2012 in South Africa. J. Infect. Dis..

[46] Kimura H (2017). Molecular evolution of the fusion protein (F) gene in human respiratory syncytial virus subgroup B. Infect. Genet. Evol..

[47] Kimura H (2016). Molecular evolution of the fusion protein gene in human respiratory syncytial virus subgroup A. Infect. Genet. Evol..

[48] Collins PL, Fearns R, Graham BS (2013). Respiratory syncytial virus: Virology, reverse genetics, and pathogenesis of disease. Curr. Top. Microbiol. Immunol..

[49] Hall CB (1990). Occurrence of groups A and B of respiratory syncytial virus over 15 years: Associated epidemiologic and clinical characteristics in hospitalized and ambulatory children. J. Infect. Dis..

[50] Lu R (2020). Genomic characterisation and epidemiology of 2019 novel coronavirus: Implications for virus origins and receptor binding. Lancet.

